# Quarterly Report of the Proceedings of the Epidemiological Society

**Published:** 1855-12

**Authors:** 


### QUARTERLY REPORT OF THE PROCEEDINGS OF THE EPIDEMIOLOGICAL SOCIETY.

### PAPERS READ AT ORDINARY MEETINGS.

*Monday, July* 2. "On the Practical Value of Vaccination in Hot Climates." By E. C. Seaton, M.D.

*Monday, August* 6. "On the Bowel Diseases of the Crimean Expedition." By W. Smart, M.D., Surgeon H.M.S. *Diamond.*—"On Cholera in Frankfort." By Dr. Varrentrapp.—"On the Premonitory Diarrhœa of Cholera." By George Todd, Esq., of West Auckland. These papers were read by Dr. McWilliam.

### DONATIONS.

Donations of books have been received from the Swedish Consul, Dr. Tholozan, Dr. Sayer, M. Payen, Dr. W. Lewis, and Dr. Howell.

### NEW MEMBERS.

*Resident.* S. W. Jones, Esq., 16, Chester-terrace, Regent's-park. Dr. J. M. McDonnell, Dublin. D. Fullerton, Esq., Randcomb-park.

*Non-resident.* G. F. Bloxsome, Esq., Southsea. P. B. Ayres, M.D., Wandsworth-road. T. Bickerton, Esq., Liverpool. J. Vincent, M.D., East Dereham, Norfolk. A. Haviland, Esq., Bridgwater.

### CORRESPONDING MEMBERS.

M. A. Trebuchet, Paris. Professor Virchow, Wurzburg. Dr. Tholozan, Paris. D. Mackinder, M.D., Gainsborough. Professor Naumann, Bonn. Dr. Kœnig, Berlin.

### DEATHS OF MEMBERS.

The members of the Society have deeply to regret the death of C. R. Walsh, Esq., their Foreign Secretary for Italy and Greece, and one of their most zealous associates.

